# P-2193. Epidemiology and healthcare resource utilization associated with Respiratory Syncytial Virus (RSV), Human Metapneumovirus (hMPV) and Parainfluenza Virus (PIV) infection in Central New York, USA

**DOI:** 10.1093/ofid/ofaf695.2356

**Published:** 2026-01-11

**Authors:** Stephen J Thomas, Dongliang Wang, Joy A Higuchi, Kelley Mooney, Rachael Cavelli, Jacqueline Malay, Michelle Klick, Samantha Gallup, Danning Huang, Oliver Martyn

**Affiliations:** Global Health Institute, SUNY Upstate Medical University, Syracuse, New York; SUNY Upstate Medical University, Syracuse, New York; Global Health Institute, SUNY Upstate Medical University, Syracuse, New York; Global Health Institute, SUNY Upstate Medical University, Syracuse, New York; Global Health Institute, SUNY Upstate Medical University, Syracuse, New York; Global Health Institute, SUNY Upstate Medical University, Syracuse, New York; Global Health Institute, SUNY Upstate Medical University, Syracuse, New York; Global Health Institute, SUNY Upstate Medical University, Syracuse, New York; Public Health and Preventative Medicine, SUNY Upstate Medical University, Syracuse, New York; Sanofi Vaccines, Copenhagen, Hovedstaden, Denmark

## Abstract

**Background:**

Respiratory viruses such as human metapneumovirus (hMPV), respiratory syncytial virus (RSV), and parainfluenza virus (PIV) are significant pathogens associated with respiratory infections, particularly in older adults and those with medical co-morbidities. These infections can lead to severe outcomes, including respiratory failure, exacerbation of chronic diseases, and increased mortality. Despite their impact, comprehensive data on the clinical features and healthcare utilization associated with these viruses in older adults is limited owing to a lack of widespread testing and diagnosis, especially so for hMPV and PIV.

**Table 1.**
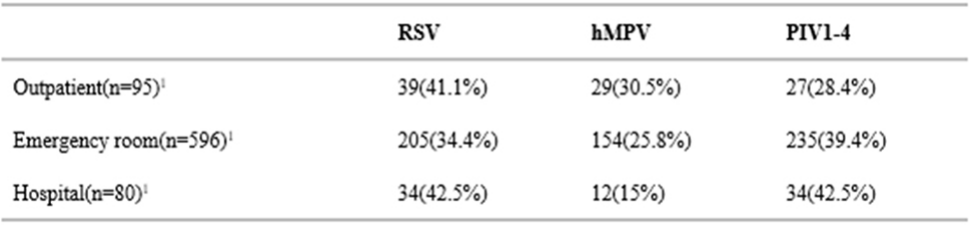
The total number of outpatient, ER and hospital were calculated by counting outpt_dx_visit_not_applic==0, er_not_applic==0, and inpt_no_applic==0, respectively. Totals include coinfections, the values under each column represent mono-infections.

**Methods:**

This retrospective chart review analyzes the clinical features and healthcare utilization of adults aged 50 years and older diagnosed with hMPV, RSV, and PIV 1-4 in Central New York State between 2021 and 2024. SUNY Upstate Medical University uses the Biofire multiplex platform, capable of detecting over 15 respiratory pathogens, on all respiratory samples. The study includes patients with positive test results for hMPV, RSV, or PIV 1-4 obtained in outpatient, emergency room and hospital settings. Key data points collected include positivity rates, demographics, comorbidities, healthcare encounters, and clinical outcomes. Statistical analyses are performed using R.

**Results:**

Positivity by care setting and pathogen over the study period are presented in table 1. Analysis of clinical characteristics, healthcare utilization and outcomes are ongoing.

**Conclusion:**

Preliminary results suggest similar rates of RSV, hMPV and PIV1-4 over the study period, although hMPV was diagnosed less frequently in the hospital setting. Analyses are ongoing and full results are expected by Q3 2025. Enhanced understanding of these infections can inform future public health strategies and vaccine development.

**Disclosures:**

Stephen J. Thomas, MD, Icoavax: Advisor/Consultant|Icoavax: Honoraria|Island Pharma: Board Member|Island Pharma: Grant/Research Support|Island Pharma: Stocks/Bonds (Public Company)|Merck: Advisor/Consultant|Merck: Grant/Research Support|Merck: Honoraria|Merck: travel|Moderna: Advisor/Consultant|Moderna: Honoraria|Pfizer: Advisor/Consultant|Pfizer: Honoraria|Pfizer: travel|Primevax: Board Member|Primevax: Stocks/Bonds (Private Company)|Rheonix: Board Member|Rheonix: Stocks/Bonds (Private Company)|Sanofi: Advisor/Consultant|Sanofi: Grant/Research Support|Sanofi: Honoraria|Sanofi: Travel|Takeda: Advisor/Consultant|Takeda: Honoraria|Takeda: travel|Valneva: Advisor/Consultant|Vaxxinity: Advisor/Consultant|Vaxxinity: Honoraria Oliver Martyn, MPH, Sanofi: Employee

